# Endothelial-Mediated Vascular Function in Frail Older Adults: A Pilot Study

**DOI:** 10.1093/geroni/igaf122.3578

**Published:** 2025-12-31

**Authors:** Anna Dietz, Sung Gi Noh, Laura Mangone, Jacob Earp, George Kuchel, Oh Sung Kwon

**Affiliations:** University of Connecticut, University of Connecticut, Connecticut, United States; University of Connecticut, Storrs Mansfield, Connecticut, United States; University of Connecticut, Storrs Mansfield, Connecticut, United States; University of Connecticut, Storrs, Connecticut, United States; UCONN HEALTH, Farmington, Connecticut, United States; University of Connecticut, Storrs Mansfield, Connecticut, United States

## Abstract

Frailty, sarcopenic obesity, endothelial dysfunction, and cardiovascular diseases (CVD) increase with aging. We investigated relationships between frailty, body composition, and endothelial dysfunction using flow-mediated dilation (FMD) and passive limb movement (PLM)-induced hyperemia. 46 participants included Healthy Young (HY, 28 ± 4 yrs, n = 16), Healthy Old (HO, 73 ± 6 yrs, n = 21), and Frail Old (FO, 78 ± 9 yrs, n = 9). Analyses in FO were stratified by body composition. Endothelium-dependent vasodilation was assessed using FMD and PLM. Plasma nitrite and nitrate were measured as NO biomarkers, and oxidative stress was quantified. FMD decreased with age (HY: 10.42 ± 0.8%; HO: 7.45 ± 0.3%, P = 0.021) and was lowest in FO (5.31%). PLM peak LBF and AUC declined with age (HY: 692 ± 107 ml/min, 249 ± 104 ml; HO: 289 ± 92 ml/min, 128 ± 45 ml; FO: 212 ± 101 ml/min, 96 ± 38 ml). Within FO, obese individuals had lower FMD and LBF than lean counterparts. Plasma nitrite decreased (HY: 140 ± 6.1 µmol/L; HO: 117 ± 7.3 µmol/L, P = 0.04; FO: 103 ± 6.6 µmol/L). Oxidative stress increased (HY: 0.45 ± 0.3 AU; HO: 2.15 ± 0.5 AU; FO: 2.88 ± 0.5 AU). Endothelial dysfunction increased with aging, frailty, and obesity. Reduced NO bioavailability and oxidative stress likely contribute. FMD and PLM assessments may aid early detection and guide interventions, including exercise, diet, and pharmacological strategies, to preserve vascular function and reduce CVD risk in frailty.

